# Food for Invalids

**Published:** 1890-01-25

**Authors:** 


### DIETETICS.

### FOOD FOR INVALIDS.

Dr. Burney Yeo's paper on "Foods for Invalids and Infants," read at the last meeting of the British Medical Association, has been published in the *British Medical Journal*, Dec. 7th. Dr. Yeo formulates two chief rules as guides to feeding in acute disease. (1) Endeavour to utilise the food to the greatest extent for the purpose of checking waste of tissue. (2) Be careful to administer no food that cannot be readily absorbed and assimilated, recollecting that the functions of the digestive organs are greatly impaired. Owing to the interruption of normal digestion, all food should be given in a fluid form. The forms of food most generally used are milk and beef-tea, in which latter term Dr. Yeo includes meat extracts, broths, etc. The great drawback to the use of milk in acute diseases, such as fevers, is that although a fluid out of the body it becomes a solid food in the stomach or intestines. There are cases in which it is not quickly digested and absorbed, and in typhoid fever serious injury may result, the motions being largely composed of firm curd, causing much intestinal irritation. Milk given to fever patients should be diluted with water, for they require pure water in larger quantities than they usually get. Alkaline water, as Vichy or Apollinaris, is preferably recommended. Two ounces of milk and two of alkaline water every hour will give the patient two pints and a half of milk a day. Whey may be often advantageously used when milk is not readily digested. The term beef-tea is usually applied to very strong extracts of meat. Yet the digestive powers of the patients to whom these concentrated essences are given are very feeble. "It is mere slavery to routine that has perpetuated the invalid's sad restriction to milk and beef-tea." Well-made mutton, veal, and chicken broth, to which some well-strained oatmeal or barley gruel can be added, make excellent foods. There is a remarkable oversight in the feeding of cases of fever by the omission of vegetable juices. Fruit soups, or vegetable soups, may be made by boiling and straining. There is also another objection to beef-tea, for it may occasionally contain peptones, which, by passing into the general circulation, act as poisons. Lastly, Dr. Burney Yeo expresses his surprise on being told by a sister that, at a certain hospital, they were forbidden to put any salt in the food of typhoid patients, although so important to the animal economy at least in health. Now, we fully agree with almost all advanced by Dr. Burney Yeo. The older physicians held that the free administration of food in fever occasionally intensifies the febrile process, and we are quite sure that the indiscriminate administration of concentrated foods in febrile diseases is often distinctly injurious. The necessity of a sufficient supply of food may be recognised, but the sufficiency depends upon the power remaining to the digestive organs. Fever patients cannot be nursed and fed *en bloc* by routine. The quantity of food and the concentration of food must be individually estimated from the condition of each patient. With regard to peptones in beef-tea, Dr. Lauder Brunton asks "whether beef-tea may not very frequently be actually injurious, and whether the products of muscular waste, which constitute the chief portion of beef-tea, or beef essence, may not under certain circumstances be actually poisons?" This may be so. But in the many maladies for which we have used beef-tea we never had occasion to suspect such consequences.

